# Moderate-to-Severe OSA and Objective Drowsiness Are Associated with Driving-Related Accidents, but Not Subjective Sleepiness: A Simulator Study

**DOI:** 10.3390/jcm15135116

**Published:** 2026-07-01

**Authors:** Erdal Aksoy, Semih Arbatli, Yeliz Celik, Nur Yasin Peker, Baran Balcan, Yüksel Peker

**Affiliations:** 1 Department of Administration and Forensic Medicine, Koc University Hospital, 34450 Istanbul, Türkiye; eaksoy@kuh.ku.edu.tr; 2 Graduate School of Sciences and Engineering, Koc University, 34450 Istanbul, Türkiye; sarbatli@ku.edu.tr; 3 Koc University Research Center for Translational Medicine, Koc University, 34010 Istanbul, Türkiye; yecelik@ku.edu.tr; 4 Department of Mechatronics Engineering, Sakarya University of Applied Sciences, 54050 Sakarya, Türkiye; yasinpeker@subu.edu.tr; 5 Department of Pulmonary Medicine, Koc University School of Medicine, 34010 Istanbul, Türkiye; mbalcan@kuh.ku.edu.tr; 6 Department of Molecular and Clinical Medicine, Sahlgrenska Academy, University of Gothenburg, 40530 Gothenburg, Sweden; 7 Department of Clinical Sciences, Respiratory Medicine and Allergology, Faculty of Medicine, Lund University, 22100 Lund, Sweden; 8 Division of Pulmonary, Allergy, and Critical Care Medicine, University of Pittsburgh School of Medicine, Pittsburgh, PA 15213, USA

**Keywords:** obstructive sleep apnea, driving simulation, drowsiness, motor vehicle accidents, excessive daytime sleepiness, Epworth Sleepiness Scale, PERCLOS, vigilance, traffic safety

## Abstract

**Background/Objectives**: Obstructive sleep apnea (OSA) is associated with an increased risk of motor vehicle accidents, traditionally attributed to excessive daytime sleepiness (EDS). However, subjective sleepiness may be underreported and does not consistently reflect functional impairment. We aimed to examine the association between objectively measured drowsiness during simulated driving and traffic accidents in OSA, and to compare the predictive roles of objective drowsiness, subjective sleepiness, and OSA severity. **Methods**: Fifty-one male drivers underwent overnight polysomnography followed by a 50-min driving simulation. OSA severity was categorized as moderate-to-severe (AHI ≥ 15 events/h) or no/mild (AHI < 15 events/h). A frontal camera captured facial expressions. Drowsiness was quantified using eye-closure-based metrics: PERCLOS, the ratio of frames with closed eyes to total observable frames, and CLOSDUR, representing eye-closure duration. Drowsiness was defined as PERCLOS ≥ 0.3 or CLOSDUR ≥ 2 s. Drowsiness-related traffic accidents were recorded. Multivariable logistic regression models were adjusted for age, body mass index, Epworth Sleepiness Scale (ESS), and total sleep time. **Results**: Drowsiness-related traffic accidents occurred more frequently in participants with moderate-to-severe OSA than in those with no or mild OSA (72.2% vs. 40.0%, *p* = 0.030). Drowsiness duration, but not ESS, was positively associated with the number of traffic accidents (r = 0.46, *p* = 0.001). While continuous AHI was not associated with accidents, moderate-to-severe OSA was independently associated with higher accident risk (adjusted OR 6.45, 95% CI 1.43–29.13; *p* = 0.015). **Conclusions**: Driving-related accident risk in OSA was associated with objectively measured drowsiness and moderate-to-severe disease, whereas subjective sleepiness assessed by the ESS showed no significant association. These findings suggest that functional impairment, rather than self-reported symptoms, may be more relevant for identifying high-risk drivers.

## 1. Introduction

Obstructive sleep apnea (OSA) is a common and heterogeneous sleep-related breathing disorder resulting from complex interactions among anatomical, physiological, genetic, and neurobiological factors that predispose patients to recurrent upper airway collapse during sleep [1,2]. These events contribute to intermittent hypoxemia, sleep fragmentation, and increased sympathetic activation, which are thought to mediate many of the adverse health consequences associated with OSA [3]. In addition to its well-established associations with cardiovascular and metabolic morbidity, OSA has important implications for public safety, particularly with regard to motor vehicle accidents [4]. Excessive daytime sleepiness (EDS) has traditionally been considered the principal mechanism linking OSA to impaired vigilance and driving performance.

Epidemiological studies have consistently shown that individuals with OSA are at increased risk of traffic accidents compared with the general population [5,6]. However, the mechanisms underlying this association remain incompletely understood. In clinical practice, subjective sleepiness—most commonly assessed by the Epworth Sleepiness Scale (ESS) [7]—is frequently used for risk stratification. Yet, ESS correlates only modestly with objective indices of OSA severity such as the apnea–hypopnea index (AHI), and many patients with moderate-to-severe OSA do not report significant daytime sleepiness. Importantly, subjective sleepiness may also be underreported, particularly among active or professional drivers, due to concerns about potential driving restrictions or loss of licensure, thereby limiting its reliability in real-world risk assessment.

Beyond EDS, several pathophysiological mechanisms may contribute to impaired driving performance in OSA. Recurrent intermittent hypoxemia, sleep fragmentation, and repeated arousals may adversely affect sustained attention, executive function, psychomotor vigilance, and reaction time, all of which are critical for safe driving performance. Experimental studies have shown that patients with OSA may exhibit delayed reaction times, impaired lane control, reduced situational awareness, and increased attentional lapses even in the absence of marked subjective sleepiness [5]. These observations suggest that functional impairment relevant to driving safety may not be adequately captured by symptom-based questionnaires alone.

This discrepancy highlights the need for objective markers that more accurately reflect functional impairment relevant to driving safety. Eye-closure-based metrics, such as the percentage of eyelid closure over time (PERCLOS) and eye-closure duration (CLOSDUR), have emerged as validated indicators of drowsiness and reduced alertness in experimental settings [8]. These measures provide continuous, real-time assessment of vigilance and may better capture transient lapses in attention that contribute to accident risk, independent of self-reported symptoms.

Despite increasing interest in objective drowsiness measures, data directly linking these metrics to accident occurrence in patients with OSA are limited. In particular, it remains unclear whether objectively quantified drowsiness is associated with accident-related outcomes beyond traditional clinical measures such as ESS or AHI. Addressing this gap is essential for improving risk stratification and informing clinical and regulatory decision-making.

Patients with OSA represent a particularly important population from a medico-legal perspective because impaired vigilance and excessive daytime sleepiness may directly affect driving safety and accident risk. As a result, many national and international regulations incorporate sleepiness assessments into fitness-to-drive evaluations for patients with OSA [9]. However, medico-legal consequences such as concerns regarding professional licensing, employment status, or driving restrictions may influence symptom reporting and reduce the reliability of subjective sleepiness measures in real-world settings. These challenges have increased interest in objective markers of vigilance and drowsiness that may provide a more accurate assessment of driving-related functional impairment.

In recent years, increasing attention has focused on objective physiological and behavioral markers of vigilance impairment in OSA. Eye-closure-based metrics derived from facial video analysis provide continuous monitoring of alertness and may detect transient reductions in vigilance that precede impaired driving performance or accident occurrence [8]. Such approaches may be particularly valuable in populations where subjective reporting of sleepiness is unreliable or intentionally minimized. However, evidence directly linking objective drowsiness metrics to accident-related outcomes in OSA remains limited.

The present study represents a secondary analysis of a previously established driving simulator cohort. The primary report from this cohort by Minhas et al. [10] focused on the development and validation of an objective drowsiness detection framework by integrating visual-based measures (PERCLOS and CLOSDUR) with synchronized electroencephalographic (EEG) features to improve the reliability of drowsiness detection.

Earlier clinical studies from the same cohort examined subjective sleepiness responses during simulator driving using the Epworth Sleepiness Scale and Karolinska Sleepiness Scale [11], as well as the diagnostic performance of the European Obstructive Sleep Apnea Screening (EUROSAS) questionnaire in professional drivers [12]. Subsequent engineering-oriented analyses further investigated EEG signal processing, microsleep identification, and optimization of EEG channel selection for drowsiness detection [13,14]. Although these studies included partially overlapping participants, they addressed distinct methodological and engineering objectives and did not examine the clinical associations among objectively measured drowsiness, OSA severity, subjective sleepiness, and simulator-recorded drowsiness-related traffic accidents. In contrast, the present study is the first to investigate these clinically relevant associations within this cohort.

Previous simulator-based and eye-tracking studies have demonstrated the feasibility of objectively detecting drowsiness during driving tasks. However, whether objectively measured drowsiness is associated with driving-related accident risk in individuals with OSA, and whether such measures provide information beyond conventional indicators such as OSA severity and subjective sleepiness, remains insufficiently understood. Addressing this knowledge gap may improve our understanding of the mechanisms linking OSA to driving impairment and accident risk.

To our knowledge, no previous study has simultaneously examined objectively measured drowsiness, OSA severity, subjective sleepiness, and simulator-based accident outcomes within the same cohort.

Therefore, the aim of the present study was to investigate the associations among OSA severity, objectively measured drowsiness, subjective sleepiness assessed by the ESS, and simulator-based driving accidents in professional drivers undergoing overnight polysomnography.

## 2. Materials and Methods

### 2.1. Study Population

This study comprised 51 professional male drivers included in a study on driving simulation conducted in the Koç University Hospital Sleep Laboratory, Istanbul. Each participant provided written informed consent and was asked to fill out questionnaires before undergoing an overnight in-hospital polysomnography (PSG). The inclusion criteria were holding a driving license longer than 3 years and having been driving actively for at least 6 or 7 days per week. Each subject was invited to voluntarily participate in the current research. The study protocol was approved by the Koç University Institutional Review Board (IRB No: 2020.292.IRB2.083) on 19 June 2020. The patient enrollment period was between 19 November 2020 and 27 March 2021. All participants provided written informed consent, and the study adhered to the Declaration of Helsinki.

Participants were recruited consecutively from patients referred for evaluation of suspected sleep-disordered breathing. All participants underwent clinical assessment, including medical history, anthropometric measurements, and standardized sleep questionnaires, before overnight polysomnography. To minimize potential confounding effects on vigilance and driving performance, individuals with unstable cardiovascular disease, active neurological disorders, severe psychiatric illness, alcohol or substance abuse, or medications known to substantially impair alertness were not included. Information regarding medication use was collected at study entry. Eleven participants (21.6%) were receiving antihypertensive treatment and six (11.8%) were receiving antidiabetic medication. No participant reported the use of sedative or hypnotic medications.

### 2.2. Sleep Assessment and Definition of OSA

All participants underwent full-night attended polysomnography (PSG) using a standardized system including electroencephalography, electrooculography, electromyography, respiratory effort signals, airflow, and oxygen saturation monitoring. Sleep and respiratory events were scored according to the American Academy of Sleep Medicine criteria [15].

Obstructive apnea was defined as a ≥90% reduction in airflow for at least 10 s in the presence of ongoing respiratory effort, and hypopnea according to standard criteria. The apnea–hypopnea index (AHI) was calculated as the number of events per hour of sleep. Participants were stratified according to OSA severity into two groups: moderate-to-severe OSA (AHI ≥ 15 events/h) and no or mild OSA (AHI < 15 events/h), in line with established clinical thresholds.

### 2.3. Subjective Sleepiness

Subjective daytime sleepiness was assessed using the Epworth Sleepiness Scale (ESS), a self-administered questionnaire evaluating the likelihood of dozing in common daily situations [7]. Scores range from 0 to 24, with higher values indicating greater sleep propensity. The ESS reflects habitual sleepiness over recent weeks rather than momentary alertness.

### 2.4. Experimental Setup and Driving Simulation

**Figure 1:**
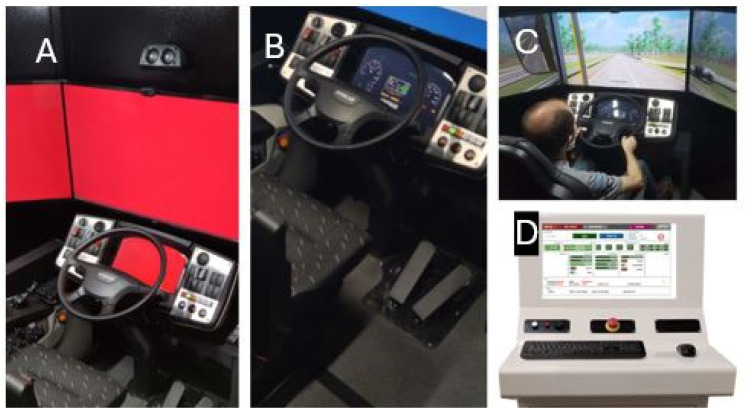
Driving simulator platform and experimental setup: (**A**) Interior view of the simulator cabin showing the immersive projection screens providing a wide field-of-view visual environment designed to replicate a monotonous highway driving scenario; (**B**) Full-scale driver cockpit including steering wheel, pedals, and dashboard interface; (**C**) Participant performing the simulated driving task within the experimental setup; (**D**) Simulator control and monitoring unit used for real-time acquisition and storage of driving performance data. The system was installed in a soundproof environment and integrated with a frontal camera for continuous facial recording, enabling objective assessment of drowsiness using adaptive eye-closure-based metrics. Driving-related events, including lane deviation, steering behavior, off-road episodes, and traffic accidents, were recorded in a time-synchronized manner. The technical details of the simulator platform and data acquisition framework have been described previously [10,13,14].

The technical details of the simulator platform, synchronized recording system, and visual/EEG-based drowsiness detection framework have been reported previously [9,10]. Briefly, the XBUS PRO Driver Training Simulator (ANGRUP Co., Tuzla, Türkiye) was used in a soundproof cabin at Koç University Hospital Sleep Laboratory (Figure 1). The simulator provides a 135-degree field of view and records driving-related events, including throttle and brake use, lane deviation, out-of-road episodes, steering irregularities, and traffic accidents. The simulated scenario consisted of a monotonous two-way highway route with low traffic density, designed to facilitate drowsiness during sustained driving. This setup was previously used for synchronized EEG–simulator analyses of microsleep and driving attributes in the same OSA cohort [10,13,14].

The driving simulation protocol was specifically designed to reproduce conditions known to promote vigilance decline during prolonged monotonous driving. Participants were instructed to maintain lane position and adhere to simulated traffic rules throughout the session. Environmental stimuli and traffic density were intentionally minimized to facilitate sustained-attention demands and increase susceptibility to drowsiness-related performance deterioration.

Participants completed a 50-min simulated driving session scheduled between 08:00 and 10:00 a.m. after overnight polysomnography. Before the test, each driver received a 10-min familiarization session with the simulator controls and driving scenario. During the experiment, a frontal camera continuously recorded facial expressions, while the simulator recorded time-stamped driving events. In related work from the same experimental platform, facial video recordings were analyzed using an adaptive Eye Aspect Ratio (EAR)-based image-processing framework to derive PERCLOS and eye-closure duration metrics, and these visual-based drowsiness episodes were validated against synchronous EEG patterns [16].

### 2.5. Objective Assessment of Drowsiness

Objective drowsiness assessment was performed using an adaptive EAR-based image-processing framework. Because fixed EAR thresholds may be affected by inter-individual anatomical differences, illumination variability, eyewear, facial expressions, and head movements, a dynamic thresholding strategy was implemented instead of a single static cutoff. To reduce noise and abrupt fluctuations, the EAR signal was processed using a median filter (window length = 17) followed by a moving average filter (window length = 5). Subsequently, the threshold was continuously updated by applying a constant shift value (0.04) to the filtered EAR signal, enabling frame-level classification of the eyes as open or closed. This adaptive approach improved the robustness of eye-closure detection under varying participant and environmental conditions.

Eye-closure-based metrics were quantified throughout the driving session, including PERCLOS, defined as the proportion of time with closed eyes relative to the total observable time, and CLOSDUR, representing the duration of individual eye-closure events. Drowsiness episodes were defined as PERCLOS ≥ 0.30 or eye closure lasting ≥2 s. These thresholds were selected to identify periods of reduced vigilance associated with impaired driving performance. Eye status was analyzed frame-by-frame using automated image-processing techniques, generating continuous temporal measures of ocular behavior during the simulation period. These ocular metrics have previously demonstrated strong associations with physiological markers of reduced vigilance and impaired driving performance [10,13].

### 2.6. Outcome Definition

The primary outcome was the occurrence of at least one drowsiness-related traffic accident (DRTA) during the 50-min simulated driving session. Off-road events were reported descriptively, whereas other simulator-derived performance measures were not analyzed in the current manuscript. Objectively detected drowsiness episodes were identified using the adaptive eye-closure-based algorithm described above. A traffic accident was classified as drowsiness-related if it occurred during an objectively detected drowsiness episode or within 30 s following the episode. Traffic accidents occurring outside this predefined temporal window were classified as unrelated to drowsiness. Thus, the primary outcome reflected simulator-recorded traffic accidents temporally associated with objective reductions in vigilance rather than all simulator accidents.

### 2.7. Relationship with Previous Publications

The present study was conducted within an ongoing driving-simulation research project involving professional male drivers evaluated for suspected sleep-disordered breathing. Previous reports from this cohort addressed distinct research questions, including subjective daytime sleepiness and simulator-induced changes in sleepiness ratings [11], validation of the European Obstructive Sleep Apnea Screening (EUROSAS) questionnaire [12], and development of EEG-validated visual drowsiness detection algorithms [10]. The current analysis is novel in focusing on the association between objectively quantified drowsiness, OSA severity, and drowsiness-related traffic accidents during simulated driving. Although there is partial overlap in participants and experimental procedures, none of the accident-related outcomes or analyses presented in the current manuscript have been reported previously.

### 2.8. Statistical Analysis

Continuous variables were assessed for normality using visual inspection of histograms and distribution characteristics. Normally distributed variables are presented as mean ± standard deviation (SD), whereas skewed variables are expressed as median and interquartile range (IQR). Categorical variables are summarized as counts and percentages.

Comparisons between participants with no/mild OSA (AHI < 15 events/h) and moderate-to-severe OSA (AHI ≥ 15 events/h) were performed using the independent-samples t test or Mann–Whitney U test for continuous variables, as appropriate, and the χ^2^ test or Fisher’s exact test for categorical variables.

Correlations between objective drowsiness duration, AHI, ESS scores, and the number of drowsiness-related traffic accidents were evaluated using Spearman correlation analyses because of the non-normal distribution of accident-related variables. Scatter plots with fitted regression lines and locally weighted smoothing (LOWESS) curves were used for visualization of continuous associations.

Multivariable logistic regression analyses were performed to examine the independent association between moderate-to-severe OSA and the occurrence of at least one drowsiness-related traffic accident during simulation. Covariates included age, BMI, ESS score, and total sleep time. Odds ratios (ORs) with 95% confidence intervals (CIs) were calculated. Additional exploratory analyses evaluated the associations of continuous AHI and ESS scores with accident-related outcomes.

To assess the robustness of the regression estimates, a nonparametric bootstrap procedure with 2000 resamples was performed for the primary multivariable logistic regression model. Bootstrap-derived Ors and 95% CIs were calculated for the association between moderate-to-severe OSA and DRTA. In addition, a Firth penalized logistic regression analysis was performed using the same covariates as in the primary model to reduce the potential influence of sparse-data bias and small-sample effects.

A two-sided *p*-value < 0.05 was considered statistically significant. Statistical analyses were performed using IBM SPSS Statistics version 28.0 (IBM Corp., Armonk, NY, USA). Figures were generated using Python 3.10 (Python Software Foundation, Wilmington, DE, USA).

## 3. Results

### 3.1. Characteristics of the Study Population

A total of 51 male drivers were included in the final analysis, with a mean age of 47.4 ± 7.4 years. Based on the predefined AHI threshold, 36 participants (70.6%) were classified as having moderate-to-severe OSA (AHI ≥ 15 events/h), whereas 15 participants (29.4%) were categorized as having no or mild OSA (AHI < 15 events/h).

Baseline demographic and sleep-related characteristics are summarized in Table 1. The two groups were generally comparable with respect to age, body mass index (BMI), total sleep time, and sleep efficiency. As expected, participants with moderate-to-severe OSA exhibited substantially higher AHI values than those with no or mild OSA. Subjective daytime sleepiness assessed by the Epworth Sleepiness Scale (ESS) did not differ significantly between the groups.

### 3.2. Drowsiness During Simulated Driving

Across the entire cohort, 453 objectively defined drowsiness episodes were identified during the 50-min simulated driving task. Participants with moderate-to-severe OSA tended to spend a greater proportion of the simulation period in a drowsy state compared with those with no or mild OSA, although the between-group difference did not reach statistical significance [median 33.2 (IQR 7.4–41.5) vs. 15.4 (2.8–42.1) minutes; *p* = 0.414].

**Figure 2:**
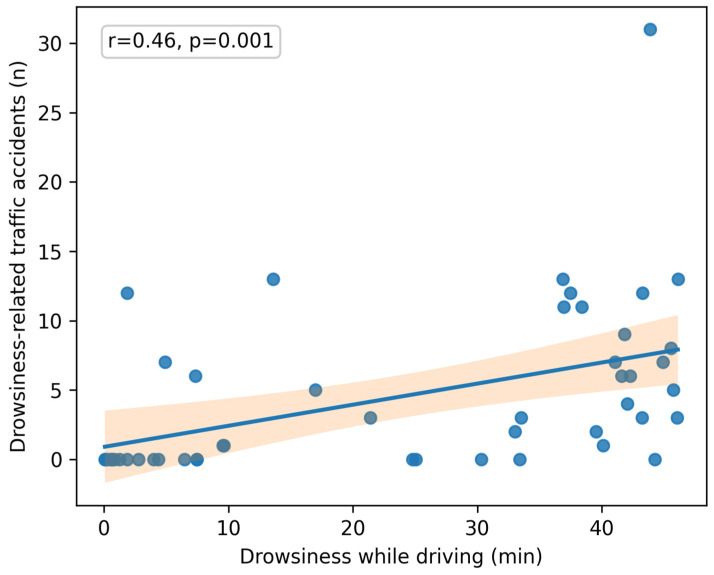
Association between objectively measured drowsiness and traffic accidents. Scatter plot showing the relationship between cumulative drowsiness duration during the simulated driving task and the number of drowsiness-related traffic accidents. The solid line represents the fitted regression line with the shaded area indicating the 95% confidence interval. A significant positive association was observed (r = 0.46, *p* = 0.001).

Objective drowsiness duration demonstrated substantial interindividual variability across the cohort. Importantly, cumulative drowsiness duration showed a significant positive correlation with the number of drowsiness-related traffic accidents (r = 0.46, *p* = 0.001) (Figure 2).

### 3.3. Subjective Sleepiness and Accident Risk

**Figure 3:**
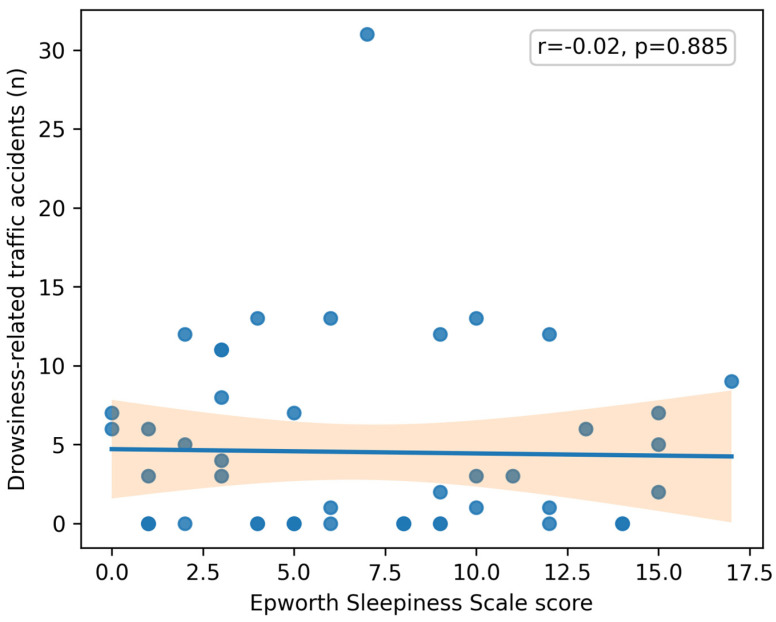
Lack of association between subjective sleepiness and traffic accidents. Scatter plot illustrating the relationship between Epworth Sleepiness Scale (ESS) scores and the number of drowsiness-related traffic accidents. The solid line represents the fitted regression line with the shaded area indicating the 95% confidence interval. No significant association was observed (r = −0.02, *p* = 0.885).

In contrast to objective drowsiness measures, subjective daytime sleepiness assessed using the ESS was not associated with accident-related outcomes. ESS scores showed no significant correlation with the number of drowsiness-related traffic accidents (r = −0.02, *p* = 0.885) (Figure 3). Similarly, ESS scores were not significantly different between participants with and without moderate-to-severe OSA.

### 3.4. OSA Severity and Drowsiness-Related Traffic Accidents

Drowsiness-related traffic accidents occurred in 6 of 15 participants (40.0%) with AHI < 15 events/h and in 26 of 36 participants (72.2%) with AHI ≥ 15 events/h *p* = 0.030). This corresponded to an absolute difference of 32.2 percentage points between the two groups. As shown in Table 2, participants with moderate-to-severe OSA experienced a higher frequency of drowsiness-related driving accidents than those with AHI < 15 events/h. The occurrence of at least one simulator-recorded accident, irrespective of its temporal relationship with drowsiness, was numerically higher among participants with moderate-to-severe OSA (88.9% vs. 73.3%), although this difference did not reach statistical significance.

To further address potential concerns regarding circularity, we performed a sensitivity analysis using the occurrence of any simulator-recorded traffic accident as the outcome. In a multivariable logistic regression model adjusted for age, BMI, and ESS, moderate-to-severe OSA (AHI ≥ 15 events/h) remained associated with accident occurrence (OR 5.98, 95% CI 0.90–39.92, *p* = 0.065). Although statistical significance was attenuated, the magnitude and direction of the association remained comparable to those observed in the primary analysis.

**Figure 4:**
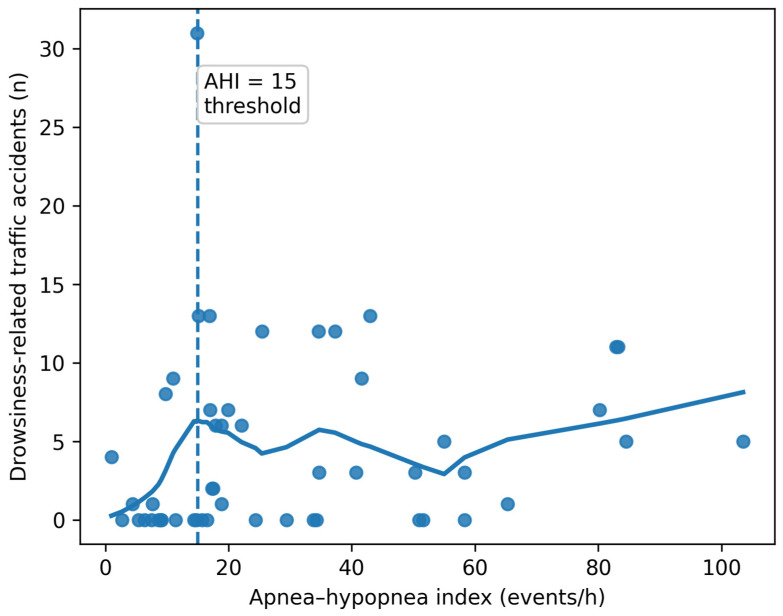
Non-linear relationship between OSA severity and traffic accidents. Scatter plot demonstrating the association between apnea–hypopnea index (AHI) and the number of drowsiness-related traffic accidents. The solid curve represents a locally weighted smoothing (LOWESS) fit, illustrating a non-linear relationship between AHI and accident risk. The dashed vertical line indicates the clinically relevant threshold for moderate-to-severe OSA (AHI = 15 events/h), highlighting a potential threshold effect rather than a linear association.

When OSA severity was evaluated as a continuous variable, AHI was not linearly associated with accident frequency (Figure 4). However, visual inspection using a locally weighted smoothing (LOWESS) curve suggested a possible nonlinear relationship, with a pattern compatible with an in-creased accident risk above the clinically established threshold for moderate-to-severe OSA (AHI ≥ 15 events/h). This observation should be considered exploratory.

### 3.5. Multivariable Analysis

**Figure 5:**
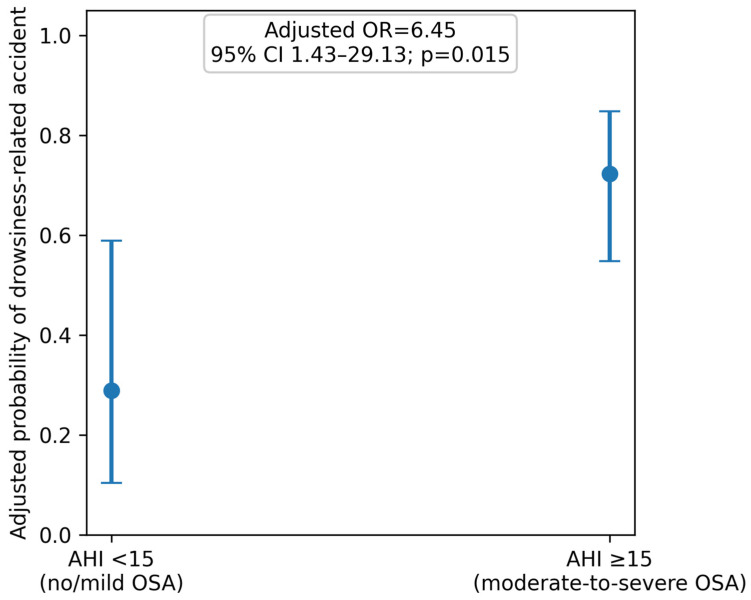
Adjusted probability of drowsiness-related accidents according to OSA severity. Estimated probability of experiencing a drowsiness-related traffic accident in participants with no/mild OSA (AHI < 15 events/h) and moderate-to-severe OSA (AHI ≥ 15 events/h). Estimates are derived from multivariable logistic regression models adjusted for age, body mass index, Epworth Sleepiness Scale score, and total sleep time. Error bars represent 95% confidence intervals. Moderate-to-severe OSA was independently associated with increased accident risk (adjusted OR 6.45, 95% CI 1.43–29.13; *p* = 0.015).

In multivariable logistic regression analyses adjusted for age, BMI, ESS score, and total sleep time, moderate-to-severe OSA remained independently associated with drowsiness-related traffic accidents (odds ratio 6.45, 95% confidence interval 1.43–29.13; *p* = 0.015) (Figure 5). To evaluate the robustness of this finding, a nonparametric bootstrap analysis with 2000 resamples was performed using the same multivariable model. The bootstrap estimates were consistent with the primary analysis, yielding a median OR of 7.74 with a bootstrap-derived 95% CI of 1.47–97.80. The primary multivariable model included 32 participants with at least one drowsiness-related traffic accident and four predictor variables (AHI category, age, BMI, and ESS), corresponding to an events-per-variable ratio of 8.0. Bootstrap analyses yielded estimates consistent with the primary model. Similarly, a Firth penalized logistic regression sensitivity analysis demonstrated a persistent association between moderate-to-severe OSA and drowsiness-related traffic accidents, although the effect estimate was modestly attenuated (OR 4.98, 95% CI 1.16–21.43).

In exploratory analyses examining continuous predictors individually, ESS was not associated with accident occurrence. Objective drowsiness duration, however, demonstrated a significant positive association with accident burden, supporting its role as a marker of impaired driving vigilance.

## 4. Discussion

In this simulator-based study of male drivers, we demonstrated that moderate-to-severe OSA was associated with a significantly higher risk of drowsiness-related traffic accidents. Importantly, objective indicators of drowsiness and OSA severity (AHI), but not subjective sleepiness assessed by the Epworth Sleepiness Scale (ESS), were associated with accident risk. These findings suggest that reliance on self-reported sleepiness alone may underestimate driving-related impairment in patients with OSA.

The relationship between OSA and increased motor vehicle accident risk has been consistently reported in epidemiological and meta-analytic studies [5,6]. Excessive daytime sleepiness (EDS) has traditionally been considered the key mediator of this association, reflecting impaired vigilance and reduced alertness. However, accumulating evidence indicates that subjective sleepiness does not fully capture the functional consequences of OSA. The modest correlation between ESS scores and objective indices of sleep-disordered breathing, as well as the presence of a “nonsleepy OSA” phenotype, highlights the limitations of questionnaire-based assessments.

Our findings extend this literature by demonstrating that ESS was not associated with accident risk in adjusted models, whereas moderate-to-severe OSA was independently associated with accident risk. In contrast, objectively measured drowsiness showed a significant unadjusted association with accident-related outcomes. This is in line with previous studies suggesting that subjective sleepiness reflects sleep propensity in specific situations rather than real-time vigilance or performance impairment [10,13]. It should also be recognized that the ESS and the objective drowsiness measures used in the present study capture different dimensions of sleepiness. Whereas the ESS reflects habitual or trait-like sleep propensity over recent weeks, eye-closure-based metrics obtained during the driving simulation reflect momentary, state-dependent reductions in vigilance. This conceptual distinction may partly explain the absence of an association between ESS scores and accident-related outcomes in the present study. Moreover, ESS may fail to identify individuals at high risk of driving-related accidents, particularly those who do not perceive or report sleepiness despite physiological vulnerability.

An important explanation for this discrepancy is the potential underreporting of sleepiness among drivers. This concern is particularly relevant in occupational drivers, because self-reported symptoms may have direct implications for fitness-to-drive decisions, professional licensing, and employment. Engleman et al. specifically demonstrated underreporting of sleepiness and driving impairment in patients with sleep apnea/hypopnea syndrome, supporting the possibility that apparently “normal” ESS scores may not reliably exclude clinically relevant sleepiness in this context [17]. Similarly, studies in commercial drivers have shown that self-reported sleepiness and symptom-based screening may poorly correspond with objective evidence of sleep-disordered breathing, suggesting that reliance on subjective reporting alone may underestimate risk [18]. In the fitness-to-drive setting, Ayeni et al. also emphasized that ESS responses may be interpreted within a medico-legal context, although their study found only limited score changes after patients were informed about licensing implications [19]. Therefore, the absence of an association between ESS and accident risk in our study may reflect not only the intrinsic limitations of ESS as a subjective measure, but also systematic reporting bias among drivers who may minimize symptoms because of perceived occupational or legal consequences [20].

Objective markers of drowsiness, particularly eye-closure-based metrics such as PERCLOS (percentage of eyelid closure), provide a more direct and continuous assessment of vigilance than subjective questionnaires [21]. Experimental and driving-simulation studies have consistently demonstrated strong associations between increased PERCLOS values, lapses in attention on psychomotor vigilance testing, impaired lane control, and deteriorated driving performance under sleep-deprived conditions [16]. Importantly, ocular metrics have been shown to correlate more closely with objective performance deficits than self-reported sleepiness, suggesting that transient fluctuations in alertness may be insufficiently captured by global subjective scales such as the ESS [22]. By detecting moment-to-moment reductions in vigilance, objective drowsiness measures may therefore better reflect the physiological mechanisms contributing to motor vehicle accidents. Our findings support the growing evidence that objective assessment of drowsiness may improve identification of high-risk individuals with OSA, particularly in populations in whom symptom reporting may be unreliable.

An important methodological consideration is the potential concern regarding circularity when objective drowsiness measures are evaluated in relation to drowsiness-related accidents. In the present study, however, objectively detected drowsiness episodes were derived from an independent facial-video-based eye-closure algorithm, whereas traffic accidents were automatically generated and recorded by the driving simulator software. Thus, the predictor and outcome variables originated from separate measurement systems, reducing the likelihood that the observed associations were driven by shared measurement processes.

An additional observation of interest was the possibility of a nonlinear relationship between OSA severity and accident risk. Although continuous AHI was not linearly associated with accident frequency, participants with moderate-to-severe OSA exhibited significantly higher accident rates than those with no or mild disease. Visual inspection of the LOWESS curve suggested a pattern compatible with a possible threshold effect around the clinically established cutoff of AHI ≥ 15 events/h. However, the present study was not specifically designed or powered to formally evaluate nonlinear associations, and this observation should therefore be considered exploratory and interpreted with caution.

From a clinical and regulatory perspective, these findings have important implications. Current driving regulations and clinical recommendations frequently rely on subjective sleepiness, particularly self-reported excessive daytime sleepiness, when assessing fitness to drive in patients with OSA [23]. However, accumulating evidence suggests that subjective sleepiness may correlate poorly with actual driving performance and crash risk, especially in individuals who underreport symptoms because of occupational or medico-legal concerns [17,19]. Several studies have demonstrated that objective measures of vigilance, attention, and sleep-disordered breathing severity may better identify drivers at increased risk of impaired performance and motor vehicle accidents than symptom-based assessments alone [5,6]. Therefore, objective vigilance monitoring and physiological markers may represent promising adjunctive tools for the assessment of driving-related functional impairment in patients with OSA. Such measures could provide complementary information beyond subjective symptom reporting; however, their clinical utility and potential role in fitness-to-drive evaluations require prospective validation in larger and more diverse populations, including studies incorporating real-world driving outcomes.

Several limitations should be acknowledged. First, the relatively small sample size and limited number of participants experiencing drowsiness-related traffic accidents resulted in wide confidence intervals around the estimated effect sizes, reducing the precision of the multivariable analyses. The primary logistic regression model included 32 participants with at least one event and four predictor variables, corresponding to an events-per-variable ratio of 8.0, which is slightly below the conventional threshold recommended for stable regression estimates. Although bootstrap resampling and Firth penalized logistic regression sensitivity analyses yielded findings broadly consistent with the primary model, some degree of sparse-data bias and model instability cannot be excluded. Therefore, the magnitude of the observed association should be interpreted cautiously and confirmed in larger independent cohorts. Second, the study population consisted exclusively of male drivers, which may limit generalizability to women and broader populations. Third, the use of a driving simulator, while providing a controlled and standardized environment, may not fully replicate real-world driving conditions. Nevertheless, simulator-based assessments have been widely used and validated for studying driving performance under conditions of sleepiness [5,6,24,25]. Fourth, subjective sleepiness was assessed using the ESS alone, and other objective measures such as the multiple sleep latency test or psychomotor vigilance testing were not performed. In addition, participation in the simulator study was voluntary and recruitment was performed within a sleep laboratory-based research cohort of professional drivers referred for evaluation of suspected sleep-disordered breathing. Therefore, the possibility of selection bias cannot be excluded. Furthermore, the primary outcome was based on a predefined classification of drowsiness-related traffic accidents occurring during or within 30 s of an objectively detected drowsiness episode. Although additional sensitivity analyses using all simulator-recorded accidents yielded broadly consistent results, alternative temporal windows could not be evaluated because event-level timestamps were not retained in the final analytical dataset. In addition, lane-departure events were not recorded as a separate simulator outcome and were therefore unavailable for analysis. Finally, the present study did not evaluate longitudinal outcomes, treatment effects, or real-world crash data. Therefore, objective drowsiness measures should be considered promising adjunctive markers of driving-related functional impairment rather than replacements for established fitness-to-drive assessments. Future prospective studies incorporating real-world driving outcomes are needed to determine their potential clinical and regulatory utility.

Despite these limitations, our study has notable strengths, including the use of polysomnography, objective drowsiness metrics, and time-resolved accident recording within a standardized driving paradigm. This integrative approach allowed us to directly link physiological vulnerability, behavioral impairment, and safety outcomes.

The present findings also highlight the potential value of integrating objective behavioral monitoring technologies into future clinical and regulatory frameworks for driving-risk assessment in OSA. Advances in computer vision, ocular tracking, and physiological signal processing may enable more individualized and dynamic evaluation of vigilance impairment than currently achievable using symptom-based questionnaires alone.

## 5. Conclusions

In this simulator-based study of professional male drivers, objectively measured drowsiness and moderate-to-severe OSA were associated with a higher risk of driving-related accidents, whereas subjective sleepiness assessed by the ESS was not. These findings suggest that objective measures of vigilance may provide complementary information beyond self-reported sleepiness when evaluating driving-related functional impairment in OSA. The association observed with moderate-to-severe OSA, but not with continuous AHI, is compatible with a possible nonlinear relationship between OSA severity and accident risk; however, this observation should be considered exploratory and requires confirmation in larger prospective studies. Future research should determine whether objective drowsiness measures can improve risk stratification and fitness-to-drive assessments in patients with OSA.

## Figures and Tables

**Table 1:** Baseline characteristics of the study population according to OSA severity.

Variable	AHI < 15 (n = 15)	AHI ≥ 15 (n = 36)	*p*-Value
Age, years	46.3 ± 8.9	47.4 ± 6.8	0.68
BMI, kg/m^2^	29.3 ± 4.8	31.9 ± 4.2	0.08
ESS score	6.9 ± 4.7	7.5 ± 5.0	0.66
Total sleep time, min	384.2 ± 46.1	389.4 ± 47.7	0.55
Sleep efficiency, %	83.2 ± 7.8	83.9 ± 7.9	0.75
AHI, events/h	8.8 ± 4.2	39.5 ± 24.5	<0.001

Data are presented as mean ± standard deviation (SD) unless otherwise indicated. Comparisons between groups were performed using independent-samples t test or Mann–Whitney U test, as appropriate. Abbreviations: AHI, apnea–hypopnea index; BMI, body mass index; ESS, Epworth Sleepiness Scale; OSA, obstructive sleep apnea; SD, standard deviation.

**Table 2:** Drowsiness and driving outcomes.

Variable	AHI < 15 (n = 15)	AHI ≥ 15 (n = 36)	*p*-Value
Drowsiness duration (min)	20.5 ± 18.7	25.3 ± 17.1	0.410
Drowsiness-related accidents (n)	0.0 (0.0–4.0)	4.0 (0.0–8.0)	0.045
Participants with ≥1 drowsiness-related accident, n (%)	6 (40.0)	26 (72.2)	0.030
Participants with ≥1 simulator-recorded accident, n (%)	11 (73.3)	32 (88.9)	0.213
Off-road events	13.9 ± 16.8	18.3 ± 16.7	0.390

Data are presented as mean ± standard deviation (SD) or median (interquartile range), as appropriate. Between-group comparisons were performed using independent-samples t test or Mann–Whitney U test.

## Data Availability

Data collected for the study, including de-identified individual par- ticipant data, will be made available to others within 6 months of the publication of this article, as will additional related documents (study protocol, statistical analysis plan, and informed con- sent form), for academic purposes (e.g., meta-analyses), upon request to the corresponding author (yuksel.peker@lungall.gu.se).

## Abbreviations

The following abbreviations are used in this manuscript:

AHI	Apnea–Hypopnea Index
BMI	Body Mass Index
CI	Confidence Interval
CLOSDUR	Eye-Closure Duration
DRTA	Drowsiness-Related Traffic Accident
EDS	Excessive Daytime Sleepiness
EEG	Electroencephalography
ESS	Epworth Sleepiness Scale
IQR	Interquartile Range
LOWESS	Locally Weighted Scatterplot Smoothing
OR	Odds Ratio
OSA	Obstructive Sleep Apnea
PERCLOS	Percentage of Eyelid Closure Over Time
